# Gsk-3**β** Inhibitors Mimic the Cardioprotection Mediated by Ischemic Pre- and Postconditioning in Hypertensive Rats

**DOI:** 10.1155/2013/317456

**Published:** 2013-10-30

**Authors:** Luisa F. González Arbeláez, Ignacio A. Pérez Núñez, Susana M. Mosca

**Affiliations:** ^1^Fellowship of Consejo Nacional de Investigaciones Científicas y Técnicas (CONICET), Centro de Investigaciones Cardiovasculares, Facultad de Ciencias Médicas, Universidad Nacional de La Plata, 60 y 120, 1900, La Plata, Buenos Aires, Argentina; ^2^Established Investigador of CONICET, Centro de Investigaciones Cardiovasculares, Facultad de Ciencias Médicas, Universidad Nacional de La Plata, 60 y 120, 1900, La Plata, Buenos Aires, Argentina

## Abstract

The aim of this study was to examine the effects of GSK-3**β** inhibitors compared with PRE and POS in spontaneously hypertensive rats (SHR). Isolated hearts were submitted to the following protocols: IC: 45 min global ischemia (GI) and 1-hour reperfusion (R); PRE: a cycle of 5 min GI and 10 minutes of R prior to 45 min GI; POS: three cycles of 30 sec GI/30 sec R at the start of R. Other hearts received lithium chloride (LiCl) or indirubin-3′-monoxime,5-iodo-(IMI) as GSK-3**β** inhibitors. All interventions reduced the infarct size observed in IC group. The expressions of P-GSK-3**β** and P-Akt decreased in IC and were restored after PRE, POS, and GSK-3**β** inhibitors treatments. An increase of cytosolic MnSOD activity and lipid peroxidation and a decrease of GSH content observed in IC hearts were attenuated in PRE, POS, and LiCl or IMI treatments. An increase of P-GSK-3**β**/VDAC physical association and a partial recovery of mitochondrial permeability were also detected after interventions. These data show that, in SHR hearts, GSK-3**β** inhibitors mimic the cardioprotection afforded by PRE and POS and suggest that a decrease in mitochondrial permeability mediated by P-GSK-3**β**/VDAC interaction is a crucial event.

## 1. Introduction

The left ventricular hypertrophy (LVH) consequent to chronically elevated blood pressure has been frequently associated with postischemic contractile dysfunction [1]. It has been previously shown in stroke-prone spontaneously hypertensive rats (SHR-SPs) [2, 3] and recently demonstrated by us in spontaneously hypertensive rats (SHR) [4] that LVH aggravates the irreversible reperfusion injury. It was also demonstrated in our and in other laboratories that ischemic preconditioning (PRE) and postconditioning (POS) were able to decrease myocardial dysfunction [5, 6] and to limit the infarct size in hypertrophied hearts [7–9]. However, data obtained in SHR hearts by Pagliaro's group [10, 11] show that these interventions do not improve the postischemic recovery of contractility nor reduce the infarct size.

It is now recognized that formation and opening of mitochondrial permeability transition pore (mPTP) are the major determinants of cardiomyocyte death following an episode of ischemia and reperfusion [12]. The mPTP causes damage to the heart because it uncouples mitochondrias, causing them to hydrolyze rather than synthesize ATP. The resulting inhibition of electron flow might explain the increased reactive oxygen species (ROS) formation induced by mPTP opening. Since the latter event is favored by ROS [13], a vicious cycle of injury amplification is likely to be established, especially at the onset of reperfusion. 

In normotensive animals, except for some studies [14, 15], most of the research supports the notion that phosphorylation at Ser9/inhibition of GSK-3*β* is required for the cardioprotection mediated by PRE and POS [16, 17]. Protein kinases, including PI3-kinase, Akt, protein kinase A, protein kinase C, and integrin-linked kinase are implicated in Ser9 phosphorylation and inactivation of GSK-3*β* [18]. Accumulating evidence indicates that phosphoSer9-GSK-3*β* (P-GSK-3*β*)-mediated cytoprotection is achieved by an increased threshold for mPTP opening [19, 20]. The mechanism by which GSK-3*β* delays mPTP opening is unclear. It has been reported that the ability of this enzyme to interact with ANT at inner mitochondrial membrane [20] and/or to phosphorylate VDAC was demonstrated in cancer cells [21].

The P-GSK-3*β* levels have also been involved in the increased vulnerability to infarction detected in hypertrophied rabbits [22] and SHR-SPs [3]. On the other hand, the oxidative stress has been involved in the genesis of hypertension [23] and plays an important role in ischemia and reperfusion injury [24]. Indeed, an attenuation of oxidative stress may be considered as one of the cardioprotective mechanisms started up by PRE and POS [25, 26]. However, GSK-3*β* regulation, its downstream targets, and its relationship to oxidative stress in those interventions in hearts from SHR remain to be determined.

Therefore, our objective was to examine the effects of GSK-3*β* inhibitors on infarct size and oxidative stress compared to those obtained by PRE and POS in isolated hearts from SHR.

## 2. Methods

An expanded “Methods” section is available in Online Data Supplements.

### 2.1. Isolated Rat Heart

All procedures followed during this investigation conform to the Guide for the Care and Use of Laboratory Animals published by the US National Institutes of Health [27] and to the guidelines laid down by the Animal Welfare Committee of La Plata School of Medicine.

Experiments were conducted in 5-months-old SHR, which were originally derived from Charles River Breeding Farms, Wilmington, Mass. Systolic blood pressure (SBP) was measured weekly using the methods indicated in Supplementary Material available online at http://dx.doi.org/10.1155/2013/317456. Animals were anesthetized with an intraperitoneal injection of sodium pentobarbital (60 mg/kg body wt). The heart was rapidly excised and perfused by the nonrecirculating Langendorff technique, and it was paced at 280 ± 10 beats/min.

### 2.2. Experimental Protocols

**Figure 1:**
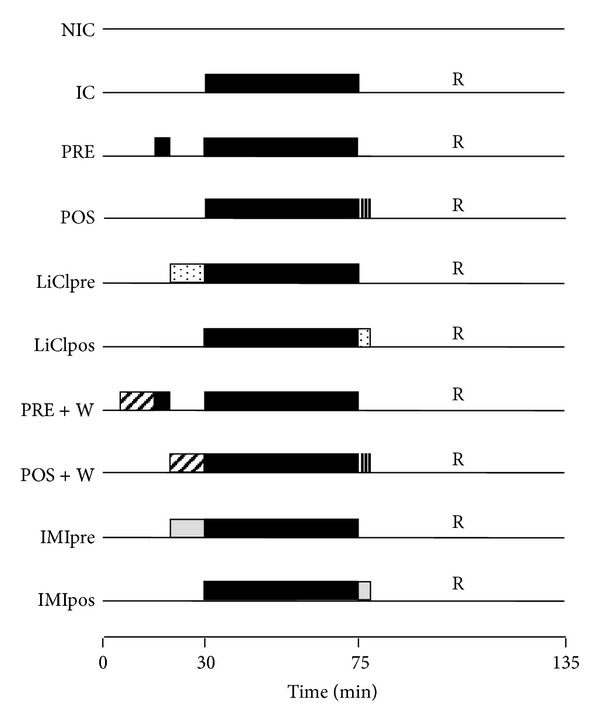
Scheme of the experimental protocols. NIC: nonischemic control; IC: ischemic control; PRE: ischemic preconditioning; POS: ischemic postconditioning; LiClpre and LiClpos: LiCl administered previously to ischemia or early during reperfusion, respectively; PRE + W: ischemic preconditioning in presence of wortmannin; POS + W: ischemic postconditioning in presence of wortmannin; IMIpre and IMIpos: IMI administered previously to ischemia or early during reperfusion, respectively.

After 30 min of stabilization, hearts from SHR were assigned to the following experimental protocols (Figure 1): nonischemic control hearts (NIC; *n* = 8): hearts were perfused for 135 min without any treatment; ischemic control hearts (IC; *n* = 10): hearts were subjected to 45 min of normothermic global ischemia followed by 1 hour of reperfusion. Global ischemia was induced by stopping the perfusate inflow line and the heart was placed in a saline bath held at 37°C; ischemic preconditioning (PRE, *n* = 12): One cycle of 5 min of ischemia and 10 min of reperfusion was applied previous to the 45 min ischemic period followed by 1-hour reperfusion; ischemic postconditioning (POS, *n* = 9): three cycles of 30 sec of ischemia and 30 sec of reperfusion was applied early during reperfusion.

Lithium chloride (LiCl) or indirubin-3′-monoxime,5-iodo- (IMI) treatment: hearts were treated with 3 mM ClLi or 1 mM IMI (GSK-3*β* inhibitors), 10 min before ischemia (LiClpre or IMIpre, *n* = 7) or during the three initial minutes of reperfusion (LiClpos or IMIpos, *n* = 7).

To assess the participation of PI3K-Akt, other hearts received wortmannin (W), PI3K inhibitor, previously to PRE and POS protocols (*n* = 7 for each other).

Separated groups of hearts subjected to the same protocols (*n* = 6 for each one) were used for biochemical determinations. Additional hearts submitted to the different protocols (*n* = 4 for each one) were used for mitochondria isolation.

### 2.3. Infarct Size Determination

Infarct size was assessed by the widely validated triphenyltetrazolium chloride (TTC) staining technique and expressed as a percentage of area at risk.

### 2.4. Systolic and Diastolic Function

Myocardial contractility was assessed by the left ventricular developed pressure (LVDP), obtained by subtracting LVEDP to LVP peak and maximal velocity of contraction (+dP/dt_max⁡_). The diastolic function was evaluated through left ventricular end diastolic pressure (LVEDP).

### 2.5. Preparation of Tissue Homogenate

At the end of reperfusion, a portion of LV was homogenized in 5 volume of 25 mmol/L PO_4_KH_2_ −140 mmol/L ClK at pH = 7.4 with a Polytron homogenizer. Aliquots of homogenate were used to assess reduced glutathione content and the remaining homogenate was centrifuged at 12000 ×g for 5 min at 4°C and the supernatant stored at −70°C for cytosolic Mn-dependent superoxide dismutase (MnSOD) activity determination.

#### 2.5.1. Lipid Peroxidation

The concentration of thiobarbituric acid reactive substances (TBARS), as an index of lipid peroxidation, was determined in the supernatant following the Buege and Aust method [28]. Absorbance at 535 nm was measured and TBARS expressed in nmol/mg of protein using an extinction coefficient of 1.56 × 10^5^ M^−1^cm^−1^. 

#### 2.5.2. Reduced Glutathione (GSH)

GSH content was determined using the Ellman's reagent [4].

#### 2.5.3. MnSOD Cytosolic Activity

SOD activity was measured by means of the nitroblue tetrazolium (NBT) method [4]. For measuring MnSOD activity, 5 mmol/L KCN was added to inhibit Cu-ZnSOD activity.

#### 2.5.4. Immunoblotting

Other portion of LV was homogenized and mitochondrial and cytosolic fractions were isolated by differential centrifugation [29]. Both fractions were resolved on SDS-PAGE, transferred to PVDF membranes, and probed with antibodies anti-PSer9 GSK-3*β*, anti-GSK-3*β*, anti-PAkt, anti-Akt, and anti-MnSOD. 

#### 2.5.5. Coimmunoprecipitation

Supernatants obtained from the first homogenization of the hearts were applied to A Sepharose protein. After centrifugation lysates were incubated with rabbit polyclonal anti-VDAC antibody and A Sepharose protein. Samples were electrophoresed and immunoblots were probed with anti-pSer9-GSK-3*β* antibody or anti-VDAC antibody.

### 2.6. Isolation of Mitochondria

Hearts were immediately removed from rats, and mitochondria from left ventricle (LV) were isolated by differential centrifugation. 

#### 2.6.1. Ca^2+^-Induced Mitochondrial Swelling

After 5 min preincubation, 0.3 mg/mL of mitochondrial suspension energized with 6 mmol/L succinate were induced to swell by the addition of CaCl_2_ 200 *μ*mol/L. These changes are observed as decreases of light scattering at excitation and emission wavelengths of 520 nm and calculated taking the difference of scattered light between before and after the addition of CaCl_2_.

### 2.7. Mitochondria Ultrastructure

A sample of mitochondrial suspension of the different experimental groups was used for ultrastructural examination using an H-600 transmission electron microscope.

### 2.8. Statistical Analysis

Data are presented as mean ± SE and repeated measures of two-way analysis of variance (ANOVA) with Newman-Keuls test were used for multiple comparisons among groups. A *P* value <0.05 was considered significant.

## 3. Results

Table 1 shows values of body weight, heart weight, heart weight/body weight ratio, and hemodynamic data of all experimental groups.

### 3.1. Infarct Size

**Figure 2:**
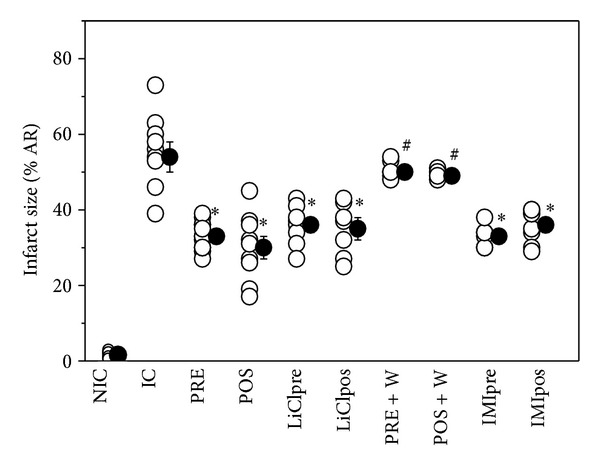
Infarct size (IS), expressed as a percentage of risk area, in nonischemic control (NIC), ischemic control (IC), preconditioned (PRE), postconditioned (POS), PRE + W (wortmannin), POS + W, lithium, and IMI treated hearts. Observe that all interventions decreased the infarct size detected in IC hearts. W abolished the protection achieved by PRE and POS. *P < 0.05 versus IC; ^#^ *P* < 0.05 versus PRE and POS.

SHR showed a SBP of 208 ± 8 mmHg. As shown in Figure 2, infarct size expressed as percentage of area at risk (AR) of ischemic control (IC) hearts was significantly reduced by PRE and POS (33 ± 1% and to 30 ± 3% versus 54 ± 4%). The treatment with LiCl or IMI before ischemia or at the beginning of reperfusion limited the infarct size at similar values to that obtained by PRE and POS. The cardioprotection exerted by PRE and POS was abolished by W treatment. W did not modify the infarct size obtained in IC group (49 ± 2%).

There was no difference in preischemic values for end-diastolic pressure and developed pressure among the nonischemic control and treated or untreated ischemic groups. Nonischemic control hearts exhibited at the end of reperfusion values of contractility similar to those observed in the stabilization period. After 45 min ischemia and 1-hour reperfusion, the recovery of contractility was scarce and reached approximately 1% of the preischemic values. This recovery was not significantly improved after all the interventions. The LVEDP (an index of diastolic stiffness) was approximately 10 mmHg at the end of the stabilization period in the different experimental groups. This parameter significantly increased reaching a value of 80 ± 10 mmHg after 1 h of reperfusion which was not modified by any intervention. 

### 3.2. Phospho-GSK-3*β* (P-GSK-3*β*) and Phospho-Akt (P-Akt) Expression

**Figure 3:**
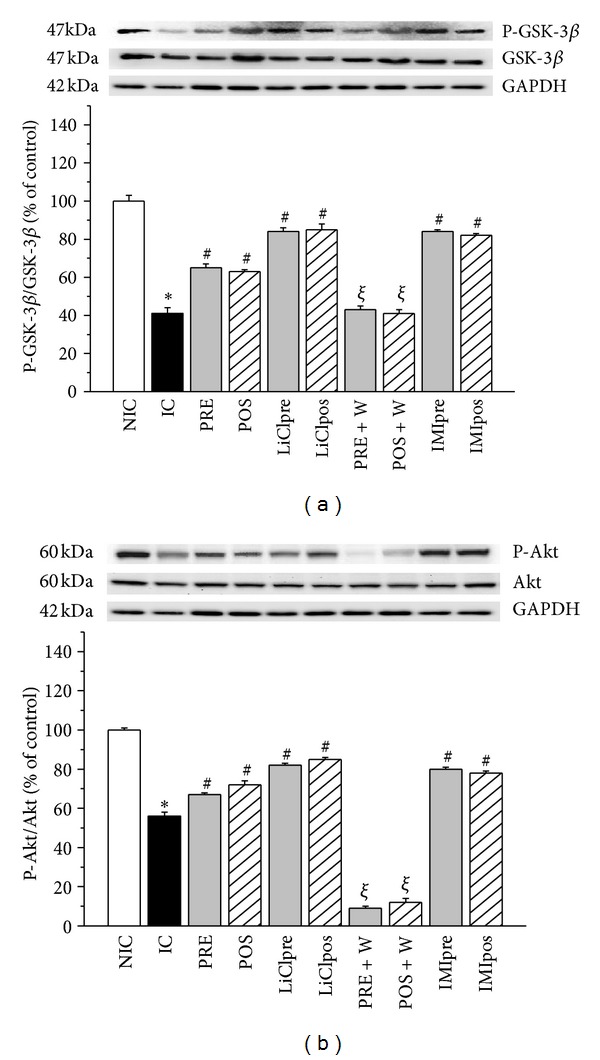
Representative immunoblots of total and phosphorylated forms and summary of densitometry data of P-GSK-3*β* (a) and phospho-Akt (P-Akt, (b)) level in cytosolic fraction in nonischemic control (NIC), ischemic control (IC), preconditioned (PRE), postconditioned (POS), PRE + W (wortmannin), POS + W, lithium, and IMI treated hearts. Immunoblots of total IC hearts showed less levels of cytosolic P-GSK-3*β*/GSK-3*β* and P-Akt/Akt ratios and they were significantly increased by all the interventions. W annulled the effects of PRE and POS. *P < 0.05 versus NIC; ^#^ *P* < 0.05 versus IC; ^*ξ*^ *P* < 0.05 versus PRE and POS.

**Figure 4:**
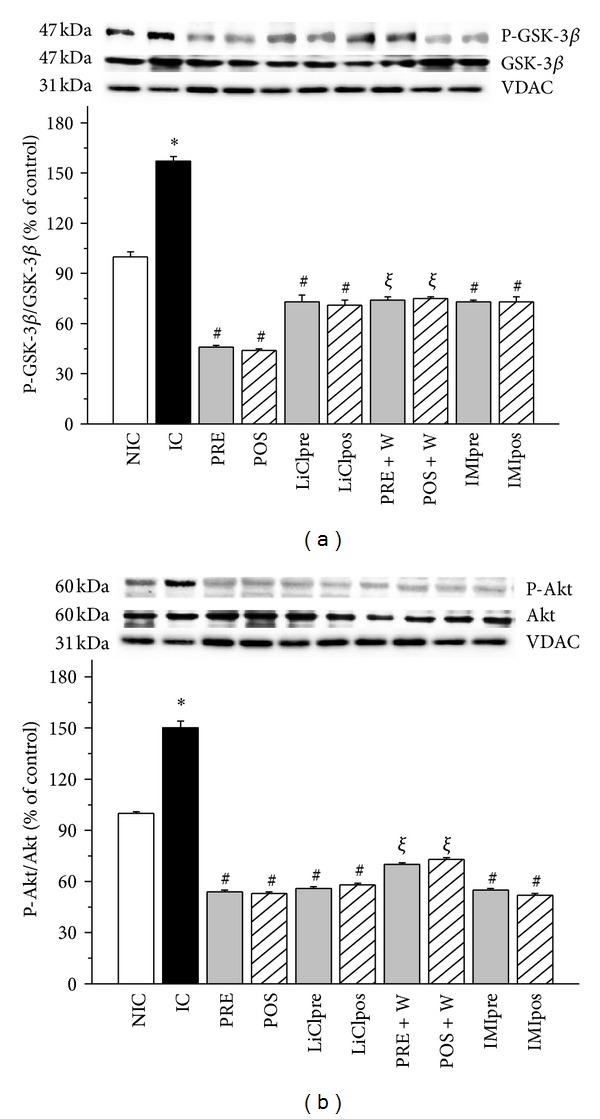
Representative immunoblots a of total and phosphorylated forms and summary of densitometry data of phospho-GSK-3*β* (P-GSK-3*β*, (a)) and phospho-Akt (P-Akt, (b)) level in mitochondrial fraction in nonischemic control (NIC), ischemic control (IC), preconditioned (PRE), postconditioned (POS), PRE + W (wortmannin), POS + W, lithium, and IMI treated hearts. VDAC was used as control. IC hearts showed high levels of cytosolic P-GSK-3*β*/GSK-3*β* and P-Akt/Akt ratios and they were significantly decreased by all the interventions. W annulled the effects of PRE and POS. *P < 0.05 versus NIC; ^#^ *P* < 0.05 versus IC; ^*ξ*^ *P* < 0.05 versus PRE and POS.

At the end of reperfusion period ischemic control hearts showed a significant decrease in P-GSK-3*β* expression in cytosolic fraction compared to nonischemic hearts (Figure 3(a)). A similar change was found in P-Akt level (Figure 3(b)). PRE and POS and LiCl, or IMI treatments increased the expression of phosphorylated forms of GSK-3*β* and Akt, showing that the GSK-3*β* inhibitors treatments have the highest values. The status of Ser9 phosphorylation in GSK-3*β* coincided with that of Akt phosphorylation, suggesting that Akt may control the activity of GSK-3*β* during ischemia and reperfusion. In W group the phosphorylation of both kinases were blunted showing P-Akt as the lowest values. When the mitochondrial fraction was analyzed the expressions of P-GSK-3*β* and P-Akt were mirror images of that found in the cytosol. Thus, an increase in ischemic hearts and levels no different or even lower than nonischemic hearts after PRE, POS, and Li, IMI or W groups were obtained (Figures 4(a) and 4(b)). W did not modify the level of phosphorylated forms of GSK-3*β* and Akt detected in IC hearts but attenuated the effects produced by PRE and POS.

### 3.3. P-GSK-3*β* and VDAC Physical Association

**Figure 5:**
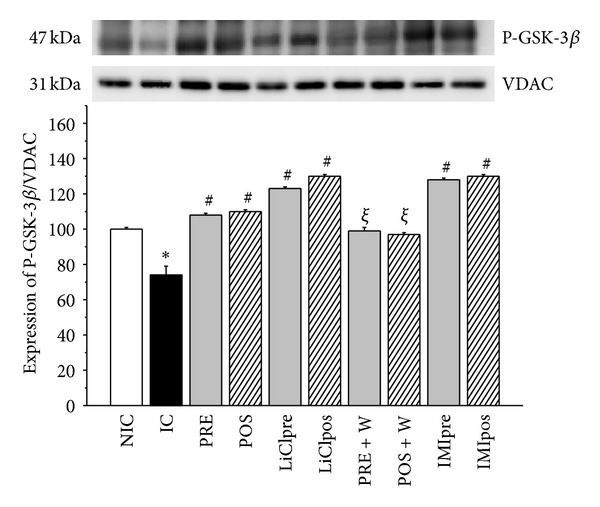
Representative immunoprecipitated and summary data of P-GSK-3*β*/VDAC and GSK-3*β*/VDAC associations in cytosolic fraction from nonischemic control (NIC), ischemic control (IC), preconditioned (PRE), postconditioned (POS), PRE + W (wortmannin), POS + W, lithium, and IMI treated hearts. These data show that the P-GSK-3*β*/VDAC association was minimal in IC hearts and maximal in hearts treated with GSK-3*β* inhibitors. W decreased significantly the P-GSK-3*β*/VDAC association detected in PRE and POS. *P < 0.05 versus NIC; ^#^ *P* < 0.05 versus IC; ^*ξ*^ *P* < 0.05 versus PRE and POS.

We assessed the physical interactions of P-GSK-3*β*/VDAC and GSK-3*β*/VDAC in the SHR heart, by coimmunoprecipitation. Cytosolic homogenates from hearts submitted to the different experimental protocols were immunoprecipitated with anti-VDAC antibody, resolved by SDS/PAGE and revealed with an anti-P-GSK-3*β* or anti-GSK-3*β* antibody. Both antibodies were able to precipitate VDAC. The cytosolic P-GSK-3*β*/VDAC association decreased in ischemic control hearts and increased in PRE, POS, and Li or IMI groups. This increase was annulled by W treatment indicating that Akt is necessary to promote that association. The total GSK-3*β*/VDAC complex was not modified by ischemia reperfusion or by interventions applied (data not shown). The anti-VDAC application demonstrated the presence of VDAC in the medium (Figure 5).

### 3.4. TBARS Concentration

**Figure 6:**
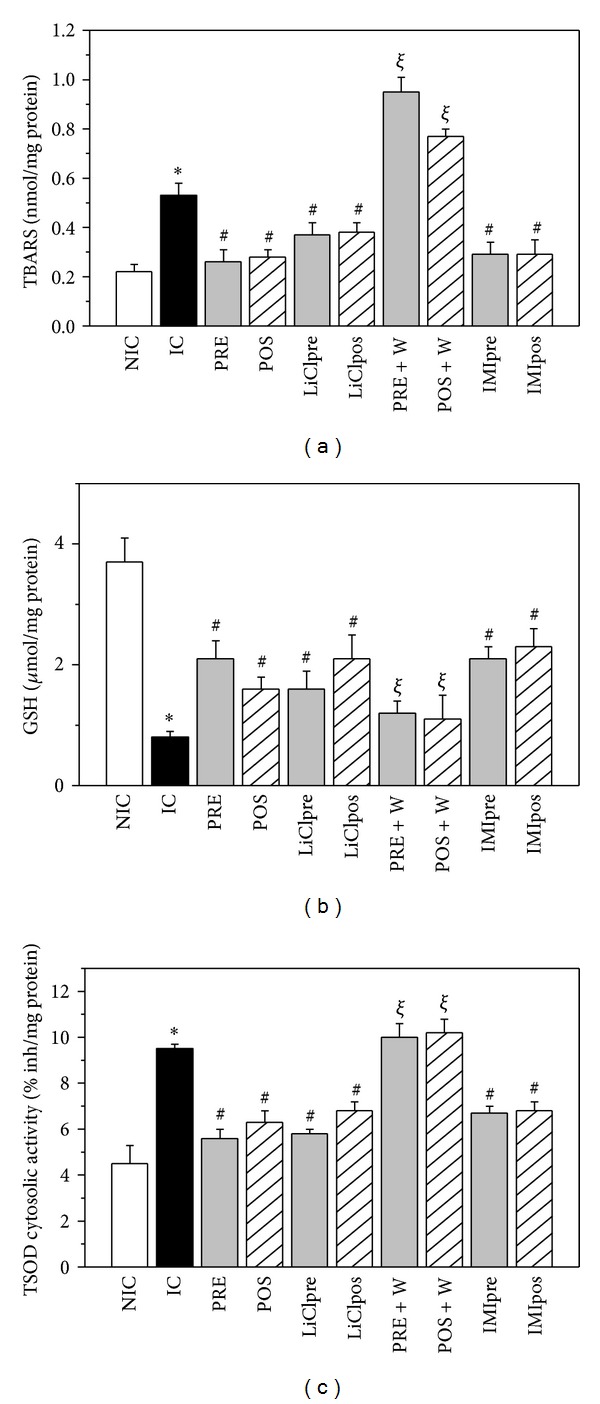
Thiobarbituric acid reactive substances concentration (TBARS, nmol/mg protein, (a)), reduced glutathione content (GSH, *μ*g/mg protein, (b)) and cytosolic total superoxide dismutase activity (TSOD, % inhibition/mg protein, (c)) in nonischemic control (NIC), ischemic control (IC), preconditioned (PRE), postconditioned (POS), PRE + W (wortmannin), POS + W, lithium, and IMI treated hearts. After ischemia and reperfusion TBARS increased, GSH levels diminished and TSOD activity increased. The interventions attenuated these changes, partially preserving the GSH content and diminishing the TSOD activity and TBARS. W reversed the changes produced by PRE and POS. *P < 0.05 versus NIC; ^#^ *P* < 0.05 versus IC; ^*ξ*^ *P* < 0.05 versus PRE and POS.

Lipid peroxidation-assessed by TBARS-increased in IC hearts and was attenuated by PRE, POS, LiCl, and IMI groups. W did not modify the TBARS content of IC hearts (0.64 ± 0.03 nmol/mg protein) but when it was added in PRE and POS protocols the increase of TBARS reached values significantly higher to that obtained in IC group (Figure 6(a)).

### 3.5. GSH Levels and Total SOD (TSOD)

GSH levels decreased in ischemic compared to nonischemic hearts and they were significantly but partially preserved by all the treatments (Figure 6(b)). TSOD cytosolic activity increased in ischemic control hearts and diminished by the different treatments and/or interventions (Figure 6(c)). W did not modify the level of GSH and TSOD cytosolic activity of IC hearts (1.11 ± 0.04 *μ*mol/mg protein and 9.20 ± 0.20% inhibition/mg protein, resp.) but reversed the changes produced by PRE and POS.

### 3.6. MnSOD Cytosolic Activity

**Figure 7:**
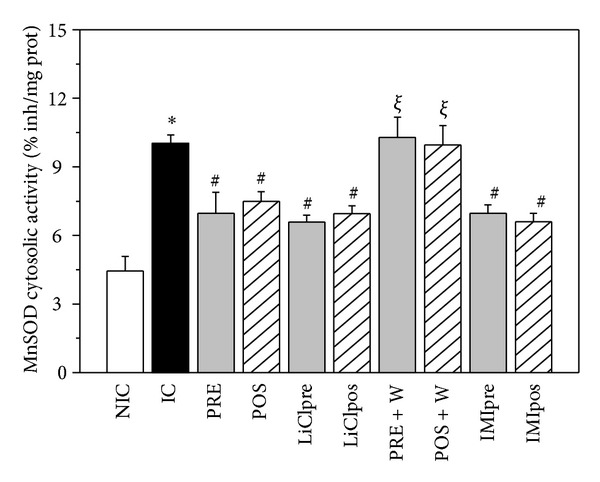
MnSOD cytosolic activity (% inhibition/mg protein) in nonischemic control (NIC), ischemic control (IC), preconditioned (PRE), postconditioned (POS), PRE + W (wortmannin), POS + W, lithium, and IMI treated hearts. Note that a significant MnSOD cytosolic activity was detected in IC hearts which was attenuated by all the interventions assessed. *P < 0.05 versus NIC; ^#^ *P* < 0.05 versus IC; ^*ξ*^ *P* < 0.05 versus PRE and POS.

MnSOD cytosolic activity significantly increased in IC hearts and the treatments attenuated this increase (Figure 7). The addition of W to IC hearts did not change MnSOD cytosolic activity (9.62 ± 0.29% inhibition/mg protein) but abolished the changes produced by PRE and POS.

### 3.7. Ca^2+^-Induced Mitochondrial Swelling

**Figure 8:**
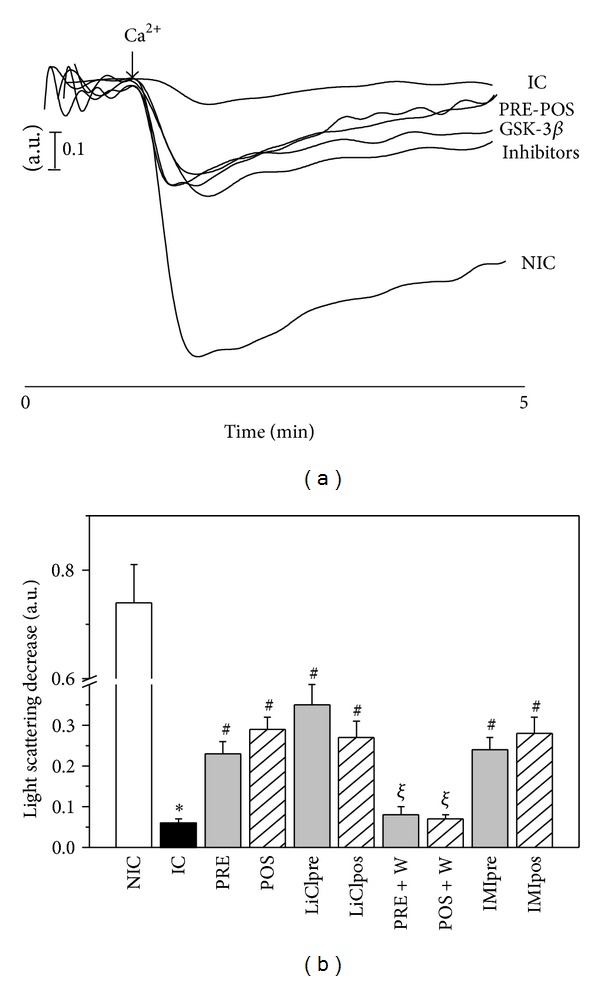
(a) Typical traces produced by 200 *μ*M Ca^2+^ addition to mitochondrial suspensions of the different experimental groups. (b) Mean values of the light scattering decreases (LSD) after Ca^2+^ addition, expressed in arbitrary units (a.u.), in nonischemic control (NIC), ischemic control (IC), preconditioned (PRE), postconditioned (POS), PRE + W (wortmannin), POS + W, lithium, and IMI treated hearts. The response of isolated mitochondria to Ca^2+^ was significantly diminished in IC hearts and was partially recovered after interventions and treatments. W abolished the favorable changes produced by PRE and POS. *P < 0.05 versus NIC; ^#^ *P* < 0.05 versus IC; ^*ξ*^ *P* < 0.05 versus PRE and POS.

Figure 8 shows typical traces of swelling experiments (Figure 8(a)) and the mean values of light scattering decrease (LSD) produced by the addition of 200 *μ*mol/L Ca^2+^ to a mitochondrial suspension from all experimental groups (Figure 8(b)). It is important to take into account that when the intermembrane connections into a mitochondrial suspension are broken, as is occurring during ischemia and reperfusion, there is a transition from an efficiently packaged particle to a state in which the inner membrane is randomly packed and the particle presents an increased cross-section to the incident light [30]. Therefore, the addition of Ca^2+^ produced lesser changes of LSD in mitochondria isolated from ischemic control hearts in comparison to those obtained from nonischemic hearts (0.06 ± 0.01 versus 0.74 ± 0.07 a.u.). W did not change the LSD obtained in IC hearts (0.05 ± 0.01 a.u.). All interventions, except PRE + W and POS + W, partially improved the LSD values.

The results of 3.6 and 3.7 items suggest that the interventions are able to diminish the proportion of permeabilized and/or damaged mitochondria produced by ischemia and reperfusion.

### 3.8. Morphology of Mitochondria

**Figure 9:**
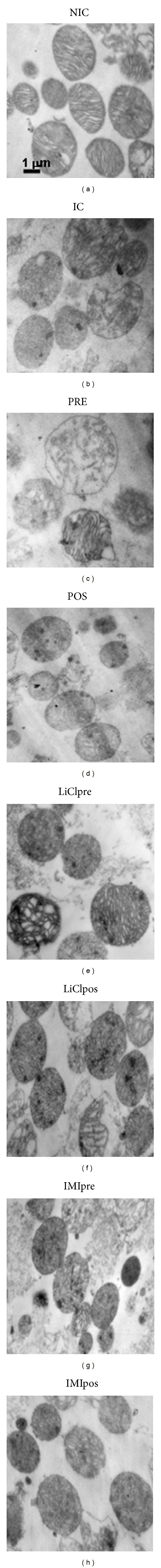
Representative electron micrographs of mitochondria obtained from nonischemic control (NIC), ischemic control (IC), preconditioned (PRE), postconditioned (POS), LiCl treated (LiClpre and LiClpos), and IMI treated (IMIpre and IMIpos) hearts. Electron microscopy confirmed the presence of mitochondria with dense cristae and intact outer membranes in NIC group while that after ischemia and reperfusion (IC) the organelle appeared swollen with damaged cristae and disrupted membranes. In intervened hearts and especially LiCl treated some mitochondria with normal morphology may be observed.

After the hearts were subjected to 45 min global ischemia and 60 min reperfusion the number of mitochondria was reduced and they appeared swollen. The cristae disappeared and vacuolar degeneration was evident. This phenomenon was lesser in the PRE, POS, LiCl, and IMI groups detecting in these hearts some mitochondria with structures relatively intact (Figure 9).

## 4. Discussion

The present study demonstrates that the physical interaction of cytosolic P-GSK-3*β* with VDAC is involved in the cardioprotective effects of PRE and POS in isolated hearts from SHR. These beneficial actions were mimicked by the treatment with an inhibitor of GSK-3*β* LiCl, evidencing the participation of this enzyme in the pathways leading to the protection. 

It has been demonstrated in normotensive animals that phosphorylation of specific serine residue (Ser9)/inhibition of GSK-3*β* plays an important role in different cardioprotective interventions [14, 31]. This importance could be attributed to the fact that GSK-3*β* is a substrate of multiple prosurvival protein kinases and that GSK-3*β* phosphorylation is therefore a step to which multiple protective signalling pathways converge. Direct inhibition of GSK-3*β* by the use of structurally different pharmacological inhibitors (SB-216763, LiCl) administered either before ischemia or reperfusion limits infarct size [32, 33]. Lithium has been shown to compete with magnesium for binding to GSK-3*β* and thereby blocks enzyme activity; then inorganic phosphate attaches to the enzyme, further inactivating it [34, 35]. In our experimental model, the treatment with both GSK-3*β* inhibitors before and after ischemia exhibited exactly the same cardioprotective profile as PRE and POS, suggesting that the infarct-limiting effect of both interventions is linked in a cause-effect relationship to GSK-3*β*-dependent mechanism. A recent study [36] shows that during prolonged ischemia activation of GSK-3*β* occurs and during the reperfusion phase an inactivation of GSK-3*β* occurs. Although “*a priori*” our results may seem opposite, some considerations might lead us to establish similarities. For example, the lesser P-GSK-3*β* expression detected after 45 min ischemia and 1 h of reperfusion could be indicating the effect of a prolonged ischemia which is not modified by 1 h of reperfusion. On the other hand, the period of ischemia (10 min) used for the protocol of ischemia reperfusion by these authors would be comparable to our PRE protocol, in which inactivation of GSK-3*β* was obtained. 

Among the kinases able to activate GSK-3*β* is the PI3K/Akt [37] which is involved in the cardioprotection against reperfusion injury [38]. The coupling of Akt and GSK-3*β* leads to inverse changes in their activities. Thus, increased Akt activity maintains the phosphorylation of GSK-3*β*, whereas decreased Akt activity leads to dephosphorylation and activation of GSK-3*β*. Our data show that a decrease of P-Akt associated to a lesser expression of P-GSK-3*β* was observed in ischemic control hearts, whereas opposite changes took place in intervened hearts. These results highlight the significant role of GSK-3*β* in ischemia and reperfusion in SHR which agree with previous papers performed in hypertensive rabbits [22] and SHR-SPs [3].

The cardioprotective interventions PRE and POS invoke the activation of signal transduction cascades by triggers which accumulate extracellularly in response to the stimulus and act on cell surface receptors or other molecular targets. These signalling pathways appear to modulate the open probability of mPTP [39]. However, a relevant piece of information is how processes occurring in the cytosol modulate mPTP opening in the inner mitochondrial membrane. It has been described that P-GSK-3*β* elevates the mPTP opening threshold [19]. Although it has been shown that critical timing of mPTP opening is within several minutes after reperfusion, we found low levels of cytosolic P-GSK-3*β* at the end of reperfusion period. However, in mitochondrial fractions of the same hearts P-GSK-3*β* expression increased. Opposite changes were detected in intervened hearts in which P-GSK-3*β* cytosolic expression increased and P-GSK-3*β* mitochondrial expression decreased. Our data in isolated mitochondria show a significant diminution of the resistance to swell by Ca^2+^ in ischemic control compared to nonischemic control hearts and a partial recovery in preconditioned, postconditioned, and LiCl or IMI treated hearts. 

The loss of internal mitochondrial membrane impermeability leads to the release of mitochondrial matrix components, as MnSOD, to cytosol. Thus, following the suggestion of a previous paper [40], the increase of cytosolic MnSOD activity detected in ischemic control hearts and a decrease observed in intervened hearts could be related to the modulation of mPTP opening as a mechanism involved in cell death. These data are supported by electron microscopy. As shown in Figure 8, mitochondria from nonischemic hearts appear dense and intact while those of ischemic-reperfused hearts are swollen and structurally more rounded. The interventions improved mitochondria structure being evident the presence of some of them intact. Thus, the changes in the structure and response to Ca^2+^ of mitochondria were accompanied by similar changes in P-GSK-3*β* cytosolic expression indicating an association between them. 

Although the mechanisms by which GSK-3*β* delays mPTP opening remains unclear some studies showed that P-GSK-3*β* interacts with ANT [20, 41] and is able to phosphorylate VDAC leading to disruption of the binding of hexokinase II and VDAC [20]. In addition Akt can directly phosphorylate hexokinase II and induce the binding of VDAC and hexokinase II [42] and thus maintain the mitochondrial integrity and promote cell survival. A VDAC phosphorylation GSK-3*β*-mediated has also been described [21, 43]. In this study, the higher levels of cytosolic P-GSK-3*β* and P-GSK-3*β*/VDAC physical association would explain the attenuation of mPTP opening as cardioprotective mechanism in intervened hearts from hypertensive animals. Previous data obtained by Costa and Garlid [44] in Sprague-Dawley rats could support our conclusion. 

According to a previous paper [45], the abrupt ROS production occurring early during reperfusion is directly correlated with depletion of the intracellular GSH pool [46]. Glutathione represents the major low-molecular weight antioxidant redox recycling thiol in mammalian cells and plays a central role in the cellular defence against oxidative damage. Other antioxidant systems such SOD are contributing to achieve the decrease in the level of ROS. In ischemic control hearts an increase of lipid peroxidation was accompanied by a depletion of GSH content and a diminution of SOD cytosolic activity. In intervened hearts lipoperoxidation decreased and GSH content and SOD activity were partially restored. These data indicate the presence of a high oxidative stress in SHR during reperfusion which was attenuated by the interventions or treatments applied and suggest that a lesser ROS production could be taking place after PRE, POS, and ClLi or IMI treatments. Considering that ROS can cause mPTP opening [47] our data in isolated mitochondria showing a partial recovery of integrity and response to Ca^2+^ in intervened hearts supports the idea that a lesser ROS production and/or release could be occurring in those conditions. A decrease of mitochondrial Ca^2+^ can also attenuate mPTP opening and thus contribute to the lesser cell death detected in intervened hearts [47]. Our data do not permit to discard this possibility.

## 5. Conclusion

The present study shows that in hearts from SHR GSK-3*β* inhibitors mimic the cardioprotective effects of PRE and POS. Therefore, our data highlight the important role of cytosolic P-GSK-3*β* and its physical association to VDAC to preserve the mitochondrial integrity via an attenuation of mPTP opening, thus, leading to a decrease in infarct size.

## Supplementary Material

In Supplementary Material we provide a detailed description of the technique of isolation of rat heart, the determination of infarct size and the biochemical reactions used to assess the antioxidant defenses and oxidative damage. Details about immunoblotting, coimmunoprecipitation, isolation of mitochondria and mitochondrial swelling Ca^2+^-induced were also added to this section.

## Figures and Tables

**Table 1:** Body weight (BW), heart weight (HW), heart weight/body weight ratio ((HW/BW)∗1000), left ventricular end diastolic pressure (LVEDP), left ventricular developed pressure (LVDP), and maximal velocity of rise of left ventricular pressure (+dP/dt_max⁡_) in all experimental groups.

Group	BW (g)	HW (g)	HW/BW	LVEDP (mmHg)	LVDP (mmHg)	+*dP*/*dt*_max⁡_ (mmHg/sec)
NIC	336 ± 12	1.50 ± 0.04	4.5 ± 0.1	10 ± 1	91 ± 1	2332 ± 14
IC	328 ± 6	1.53 ± 0.07	4.7 ± 0.2	13 ± 2	88 ± 6	2340 ± 126
PRE	329 ± 13	1.43 ± 0.04	4.4 ± 0.2	12 ± 1	87 ± 7	2318 ± 147
POS	325 ± 13	1.52 ± 0.07	4.7 ± 0.2	10 ± 1	89 ± 6	2393 ± 129
LiClpre	328 ± 5	1.46 ± 0.05	4.5 ± 0.2	12 ± 1	87 ± 4	2300 ± 98
LiClpos	325 ± 10	1.46 ± 0.03	4.5 ± 0.2	10 ± 1	91 ± 2	2333 ± 39
PRE + W	340 ± 12	1.60 ± 0.07	4.7 ± 0.2	10 ± 2	86 ± 8	2260 ± 120
POS + W	332 ± 6	1.52 ± 0.05	4.6 ± 0.2	11 ± 1	86 ± 8	2280 ± 150
IMIpre	330 ± 3	1.54 ± 0.03	4.7 ± 0.1	12 ± 1	90 ± 1	2338 ± 100
IMIpos	322 ± 10	1.52 ± 0.05	4.7 ± 0.2	11 ± 1	89 ± 4	2310 ± 130

## References

[1] Friehs I, Del Nido PJ (2003). Increased susceptibility of hypertrophied hearts to ischemic injury. *Annals of Thoracic Surgery*.

[2] Chen H, Azuma M, Maeda K, Kajimoto N, Higashino H (2000). Impaired heart function and noradrenaline release after ischaemia in stroke-prone spontaneously hypertensive rats. *Clinical and Experimental Pharmacology and Physiology*.

[3] Yano T, Miki T, Tanno M (2011). Hypertensive hypertrophied myocardium is vulnerable to infarction and refractory to erythropoietin-induced protection. *Hypertension*.

[4] Fantinelli JC, Pérez Núñez IA, González Arbeláez LF, Schinella GR, Mosca SM (2013). Participation of mitochondrial permeability transition pore in the effects of ischemic preconditioning in hypertrophied hearts: role of NO and mitoK(ATP). *International Journal of Cardiology*.

[5] Fantinelli JC, Mosca SM (2007). Comparative effects of ischemic pre and postconditioning on ischemia-reperfusion injury in spontaneously hypertensive rats (SHR). *Molecular and Cellular Biochemistry*.

[6] Ferdinandy P, Schulz R, Baxter GF (2007). Interaction of cardiovascular risk factors with myocardial ischemia/reperfusion injury, preconditioning, and postconditioning. *Pharmacological Reviews*.

[7] Speechly-Dick ME, Baxter GF, Yellon DM (1994). Ischemic preconditioning protects hypertrophied myocardium. *Cardiovascular Research*.

[8] Ebrahim Z, Yellon DM, Baxter GF (2007). Ischemic preconditioning is lost in aging hypertensive rat heart: independent effects of aging and longstanding hypertension. *Experimental Gerontology*.

[9] Dai W, Simkhovich BZ, Kloner RA (2009). Ischemic preconditioning maintains cardioprotection in aging normotensive and spontaneously hypertensive rats. *Experimental Gerontology*.

[10] Penna C, Tullio F, Moro F, Folino A, Merlino A, Pagliaro P (2010). Effects of a protocol of ischemic postconditioning and/or Captopril in hearts of normotensive and hypertensive rats. *Basic Research in Cardiology*.

[11] Penna C, Tullio F, Perrelli M-G (2011). Ischemia/reperfusion injury is increased and cardioprotection by a postconditioning protocol is lost as cardiac hypertrophy develops in nandrolone treated rats. *Basic Research in Cardiology*.

[12] Tsujimoto Y, Shimizu S (2007). Role of the mitochondrial membrane permeability transition in cell death. *Apoptosis*.

[13] Di Lisa F, Canton M, Carpi A (2011). Mitochondrial injury and protection in ischemic pre-and postconditioning. *Antioxidants and Redox Signaling*.

[14] Nishino Y, Webb IG, Davidson SM (2008). Glycogen synthase kinase-3 inactivation is not required for ischemic preconditioning or postconditioning in the mouse. *Circulation Research*.

[15] Skyschally A, van Caster P, Boengler K (2009). Ischemic postconditioning in pigs: no causal role for risk activation. *Circulation Research*.

[16] Gomez L, Paillard M, Thibault H, Derumeaux G, Ovize M (2008). Inhibition of GSK3*β* by postconditioning is required to prevent opening of the mitochondrial permeability transition pore during reperfusion. *Circulation*.

[17] Gross ER, Hsu AK, Gross GJ (2008). Delayed cardioprotection afforded by the glycogen synthase kinase 3 inhibitor SB-216763 occurs via a KATP- and MPTP-dependent mechanism at reperfusion. *The American Journal of Physiology*.

[18] Jope RS, Johnson GVW (2004). The glamour and gloom of glycogen synthase kinase-3. *Trends in Biochemical Sciences*.

[19] Juhaszova M, Zorov DB, Yaniv Y, Nuss HB, Wang S, Sollott SJ (2009). Role of glycogen synthase kinase-3*β* in cardioprotection. *Circulation Research*.

[20] Nishihara M, Miura T, Miki T (2007). Modulation of the mitochondrial permeability transition pore complex in GSK-3*β*-mediated myocardial protection. *Journal of Molecular and Cellular Cardiology*.

[21] Pastorino JG, Hoek JB, Shulga N (2005). Activation of glycogen synthase kinase 3*β* disrupts the binding of hexokinase II to mitochondria by phosphorylating voltage-dependent anion channel and potentiates chemotherapy-induced cytotoxicity. *Cancer Research*.

[22] Barillas R, Friehs I, Cao-Danh H, Martinez JF, del Nido PJ (2007). Inhibition of glycogen synthase kinase-3*β* improves tolerance to ischemia in hypertrophied hearts. *Annals of Thoracic Surgery*.

[23] Lassègue B, Griendling KK (2004). Reactive oxygen species in hypertension. *The American Journal of Hypertension*.

[24] Downey JM (1990). Free radicals and their involvement during long-term myocardial ischemia and reperfusion. *Annual Review of Physiology*.

[25] Quarrie R, Lee DS, Steinbaugh G (2012). Ischemic preconditioning preserves mitochondrial membrane potential and limits reactive oxygen species production. *Journal of Surgical Research*.

[26] Kin H, Wang N-P, Mykytenko J (2008). Inhibition of myocardial apoptosis by postconditioning is associated with attenuation of oxidative stress-mediated nuclear factor-*κ*B translocation and TNF*α* release. *Shock*.

[27] Guide for the Care and Use of Laboratory Animals.

[28] Buege JA, Aust SD (1978). Microsomal lipid peroxidation. *Methods in Enzymology*.

[29] Baines CP, Zhang J, Wang G-W (2002). Mitochondrial PKC*ε* and MAPK form signaling modules in the murine heart: enhanced mitochondrial PKC*ε*-MAPK interactions and differential MAPK activation in PKC*ε*-induced cardioprotection. *Circulation Research*.

[30] Beavis AD, Brannan RD, Garlid KD (1985). Swelling and contraction of the mitochondrial matrix. I: a structural interpretation of the relationship between light scattering and matrix volume. *Journal of Biological Chemistry*.

[31] Miura T, Miki T (2009). GSK-3*β*, a therapeutic target for cardiomyocyte protection. *Circulation Journal*.

[32] Gross ER, Hsu AK, Gross GJ (2004). Opioid-induced cardioprotection occurs via glycogen synthase kinase *β* inhibition during reperfusion in intact rat hearts. *Circulation Research*.

[33] Obame FN, Plin-Mercier C, Assaly R (2008). Cardioprotective effect of morphine and a blocker of glycogen synthase kinase 3*β*, SB216763 [3-(2,4-dichlorophenyl)-4(1-methyl-1H-indol-3-yl)-1H- pyrrole-2,5-dione], via inhibition of the mitochondrial permeability transition pore. *Journal of Pharmacology and Experimental Therapeutics*.

[34] Zhan F, Phiel CJ, Spece L, Gurvich N, Klein PS (2003). Inhibitory phosphorylation of glycogen synthase kinase-3 (GSK-3) in response to lithium: evidence for autoregulation of GSK-3. *Journal of Biological Chemistry*.

[35] Ryves WJ, Harwood AJ (2001). Lithium inhibits glycogen synthase kinase-3 by competition for magnesium. *Biochemical and Biophysical Research Communications*.

[36] Zhai P, Sciarretta S, Galeotti J, Volpe M, Sadoshima J (2011). Differential roles of gsk-3*β* during myocardial ischemia and ischemia/reperfusion. *Circulation Research*.

[37] Pap M, Cooper GM (1998). Role of glycogen synthase kinase-3 in the phosphatidylinositol 3- kinase/Akt cell survival pathway. *Journal of Biological Chemistry*.

[38] Zhu M, Feng J, Lucchinetti E (2006). Ischemic postconditioning protects remodeled myocardium via the PI3K-PKB/Akt reperfusion injury salvage kinase pathway. *Cardiovascular Research*.

[39] Hausenloy DJ, Ong S-B, Yellon DM (2009). The mitochondrial permeability transition pore as a target for preconditioning and postconditioning. *Basic Research in Cardiology*.

[40] Jin Z-Q, Zhou H-Z, Cecchini G, Gray MO, Karliner JS (2005). MnSOD in mouse heart: acute responses to ischemic preconditioning and ischemia-reperfusion injury. *The American Journal of Physiology*.

[41] Terashima Y, Sato T, Yano T (2010). Roles of phospho-GSK-3*β* in myocardial protection afforded by activation of the mitochondrial KATP channel. *Journal of Molecular and Cellular Cardiology*.

[42] Miyamoto S, Murphy AN, Brown JH (2008). Akt mediates mitochondrial protection in cardiomyocytes through phosphorylation of mitochondrial hexokinase-II. *Cell Death and Differentiation*.

[43] Das S, Wong R, Rajapakse N, Murphy E, Steenbergen C (2008). Glycogen synthase kinase 3 inhibition slows mitochondrial adenine nucleotide transport and regulates voltage-dependent anion channel phosphorylation. *Circulation Research*.

[44] Costa ADT, Garlid KD (2008). Intramitochondrial signaling: interactions among mitoKATP, PKC*ε*, ROS, and MPT. *The American Journal of Physiology*.

[45] Leichtweis S, Ji LL (2001). Glutathione deficiency intensifies ischaemia-reperfusion induced cardiac dysfunction and oxidative stress. *Acta Physiologica Scandinavica*.

[46] Slodzinski MK, Aon MA, O’Rourke B (2008). Glutathione oxidation as a trigger of mitochondrial depolarization and oscillation in intact hearts. *Journal of Molecular and Cellular Cardiology*.

[47] Halestrap AP (2009). Mitochondria and reperfusion injury of the heart-A holey death but not beyond salvation. *Journal of Bioenergetics and Biomembranes*.

