# Supplementary material for: Gsk-3β Inhibitors Mimic the Cardioprotection Mediated by Ischemic Pre- and Postconditioning in Hypertensive Rats
Source: Biomed Res Int. 2013 Oct 30;2013:317456. doi: 10.1155/2013/317456 (PMC3830772; doi:10.1155/2013/317456)
Supplement: Supplementary file 1 — In Supplementary Material we provide a detailed description of the technique of isolation of rat heart, the determination of infarct size and the biochemical reactions used to assess the antioxidant defenses and oxidative damage. Details about immunoblotting, coimmunoprecipitation, isolation of mitochondria and mitochondrial swelling Ca2+-induced were also added to this section. [file 317456.f1.pdf]

Supplementary Methods

Experiments were conducted in 5-months-old SHR, which were originally derived from Charles River Breeding Farms, Wilmington, Mass. All animals were identically housed under controlled lighting and temperature conditions with free access to standard rat chow and tap water. Beginning at 12 weeks of age, systolic blood pressure (SBP) was measured weekly in all animals by the standard tail-cuff method [1] and following the modifications detailed in a recent paper by Fritz and Rinaldi [2].

2.1. Isolated rat heart

The heart was rapidly excised and perfused by the non-recirculating Langendorff technique with Ringer's solution containing (in mmol/L): 118 NaCl, 4.7 KCl, 1.2 $MgSO_4$, 1.35 $CaCl_2$, 20 $NaCO_3H$ and 11.1dextrose. The buffer was saturated with a mixture of 95% O_2-5% CO_2, had a pH 7.4, and was maintained at 37°C. The conductive tissue in the atrial septum was damaged with a fine needle to achieve atrioventricular block, and the right ventricle was paced at 280 ± 10 beats/min. A latex balloon tied to the end of a polyethylene tube was passed into the left ventricle through the mitral valve; the opposite end of the tube was then connected to a Statham P23XL pressure transducer. The balloon was filled with water to provide an end-diastolic pressure (LVEDP) of 8-12 mmHg and this volume remained unchanged for the rest of the experiment. Coronary perfusion pressure (CPP) was monitored at the point of cannulation of the aorta and adjusted to approximately 70 mmHg. Coronary flow (CF), controlled with a peristaltic pump, was 11 ± 2 mL/min. Left

ventricular pressure (LVP) and CPP data were acquired by using an analog-to-digital converter and acquisition software (Chart V4.2.3 ADInstruments).

2.2. Infarct size determination

Infarct size was assessed by the widely validated triphenyltetrazolium chloride (TTC) staining technique [3]. At the end of reperfusion, atrial and right ventricular tissues were excised and left ventricle (VI) was frozen. The freeze VI was cut into six transverse slices, which were incubated for 5 minutes at 37°C in a 1% solution of triphenyltetrazolium chloride (TTC). To measure myocardial infarction, the slices were weighed and scanned. The infarcted (pale) and viable ischemic/reperfused (red) areas were measured by computed planimetry (Scion Image 1.62; Scion Corp., Frederick, Maryland, USA). Infarct weights were calculated as (A1 × W1) + (A2 × W2) + (A3 × W3) + (A4 × W4) + (A5 × W5) + (A6 × W6), where A is the area of infarct for the slice and W is the weight of the respective section. Infarct size was expressed as a percentage of total area (area at risk, AR) [4].

2.3. Reduced glutathione (GSH)

GSH content was determined using the Ellman´s reagent. This method was based on the reaction of GSH with 5,5′ dithiobis (2-nitrobenzoic acid) to give a compound that absorbs at 412 nm. GSH levels were expressed as μg/mg of protein.

2.4. MnSOD cytosolic activity

SOD activity was measured by means of the nitroblue tetrazolium (NBT) method. Briefly, the supernatant was added to the reaction mixture of NBT with xanthine-oxidase, and the SOD activity measured colorimetrically in the form of inhibitory activity toward blue

formazan formation by SOD in the reaction mixture. For measuring MnSOD activity, 5 mmol/l KCN was added to inhibit Cu-ZnSOD activity.

2.5.Immunoblotting

Other portion of LV was homogenized and mitochondrial and cytosolic fractions were isolated by differential centrifugation. Briefly, LV were homogenized in ice-cold RIPA buffer (300 mmol/L sacarosa, 1 mmol/L DTT, 4 mmol/L EGTA, 20 mmol/L TRIS pH 7.4, 1% Triton X, 10% protease cocktail, 25 µmol/L FNa, 1 µmol/L Ortovanadate) and centrifuged at 1000 x g for 15 minutes at 4 ºC to remove nuclei and debris. The supernatant was subsequently centrifuged at 10 000 x g for 30 min. The resultant supernatant was subsequently centrifuged at 100 000 x g for 1 hour to yield the cytosolic fraction. The 10 000 x g pellet, corresponding to the mitochondrial fraction, was resuspended and centrifuged again at 10 000g for 30 minutes. The washed mitochondria were then resuspended and homogenized. Mitochondrial (100 µg) and cytosolic proteins (60 µg) were resolved on SDS-PAGE and transferred to PVDF membrane (2 hours). Equal loading of samples was confirmed by Ponceau S staining. Membranes were blocked with 5% non-fat milk in Tris-buffered saline (pH 7.5) containing 0.1 % Tween (TBS-T), and probed overnight at 4ºC with antibodies anti–PSer9 GSK-3β (1:1000, Santa Cruz Biotechnology), anti-GSK-3β (1:1000, Santa Cruz Biotechnology) anti-PAkt (1:1000, Santa Cruz Biotechnology), anti-Akt (1:1000, Santa Cruz Biotechnology) and anti-MnSOD (1:1000, Abcam). Membranes were washed four times for 10 min in TBS-T prior to addition of anti-rabbit secondary antibody (1:5000, Santa Cruz Biotechnology) and protein bands were analyzed by a chemiluminescent system (ECL Plus; Amersham Biosciences).

GAPDH and VDAC/porin signals were used as a loading control of cytosolic and mitochondrial fractions, respectively.

2.6. Coimmunoprecipitation

Supernatants (100 µl) obtained from the first homogenization of the hearts were applied to 50 µl A Sepharose protein (50% slurry) for 1 h at 4 ºC. After centrifugation (5 min, 8000 x g, 4ºC), lysates were incubated overnight with rabbit polyclonal anti-VDAC antibody (5 µl, 6.7 mg IgG) and 100 µl A Sepharose protein (at 4ºC, overnight). Resin was washed two times to remove any nonspecifically bound proteins. After centrifugation (8000 x g) resin was resuspended in SDS-PAGE sample buffer. Samples were electrophoresed on 7.5% or 10% acrylamide gels, as indicated. Immunoblots were probed with anti-pSer9-GSK-3β antibody (1:1000 dilutions) or rabbit anti-VDAC antibody (1:1000 dilutions).

2.7. Protein determination

The protein concentration was evaluated by the Bradford method [5] using bovine serum albumin as a standard.

2.8. Isolation of rat heart mitochondria.

LV were washed and homogenized in ice-cold isolation solution (IS) consisting of 75 mM sucrose, 225 mM mannitol, and 0.01 mM EGTA neutralized with Trizma buffer at pH 7.4. After the tissue pieces were settled, the entire supernatant was discarded and fresh IS (5 ml) was added, and the mixture was transferred to a hand homogenizer. Proteinase (0.8 mg, bacterial, type XXIV, Sigma, formerly called Nagarse) was added just before starting the homogenization procedure. The whole homogenization procedure took no longer than 14 min in two steps of 7 min each (with 5 ml addition of fresh IS each). The homogenate was carefully transferred after each step to a polycarbonate centrifuge tube. After 5 min of

480×g of centrifugation to discard unbroken tissue and debris, the supernatant was centrifuged at 7 700×g for 10 min to sediment the mitochondria. The mitochondrial pellet was washed twice with IS and the last one with suspension solution (IS without EGTA) at 7,700×g for 5 min each.

2.8. Mitochondrial swelling Ca^{2+}-induced

The ability of mitochondria to resist swelling was assessed by incubating 0.3 mg/mL of isolated mitochondria in a buffer containing (in mmol/L): 120 KCl, 20 MOPS, 10 Tris HCl, and 5 KH_2PO4 adjusted to pH = 7.4. After 5-min preincubation, the mitochondria energized with the addition of 5 mmol/L succinate were induced to swell with 200 μmol/L $CaCl_2$. If mPTP is open in the presence of Ca^{2+} loading, solutes will be free to enter the inner matrix, causing the mitochondria to swell. These changes are observed as decreases of light scattering and followed using a temperature-controlled Hitachi F4500 spectrofluorometer operating with continuous stirring at excitation and emission wavelengths of 520 nm [6]. Light scattering decrease (LSD) was calculated for each sample by taking the difference of scattered light between before and after the addition of $CaCl_2$.

REFERENCES

[1] R.D. Buñag, ¨Validation in awake rats of a tail-cuff method for measuring systolic pressure¨ Journal Applied of Physiology, vol. 34, pp. 279-282, 1973.

[2] M. Fritz, and G. Rinaldi, ¨Blood pressure measurement with the tail-cuff method in Wistar and spontaneously hypertensive rats: influence of adrenergic- and nitric oxide-mediated vasomotion¨ Journal of Pharmacological and Toxicological Methods, vol. 58, pp. 215-221, 2008.

[3] M.C. Fishbein, S. Meerbaum, J. Rit, U. Lando, K. Kanmatsuse, J.C. Mercier, E. Corday, and W. Ganz, ¨Early phase acute myocardial infarct size quantification: validation of the triphenyl tetrazolium chloride tissue enzyme staining technique¨American Heart Journal, vol. 101; pp. 593-600, 1981.

[4] M. Suzuki, N. Sasaki, T. Miki, N. Sakamoto, Y. Ohmoto-Sekine, M. Tamagawa, S. Seino, E. Marbán, and H. Nakaya, ¨Role of sarcolemmal K(ATP) channels in cardioprotection against ischemia/reperfusion injury in mice¨ Journal of Clinical Investigation, vol. 109, pp. 509-516, 2002.

[5] M.M. Bradford, ¨A rapid and sensitive method for the quantitation of microgram quantities of protein utilizing the principle of protein-dye binding¨ Analytical Biochemistry, vol. 72, pp. 248-254, 1976.

[6] H.T. Facundo, J.G. de Paula, and A.J. Kowaltowski, ¨Mitochondrial ATP-sensitive K+ channels are redox-sensitive pathways that control reactive oxygen species production¨ Free Radical Biology and Medicine, vol. 42, pp. 1039-1048, 2007.
